# The Stress and Vascular Catastrophes in Newborn Rats: Mechanisms Preceding and Accompanying the Brain Hemorrhages

**DOI:** 10.3389/fphys.2016.00210

**Published:** 2016-06-14

**Authors:** Oxana Semyachkina-Glushkovskaya, Ekaterina Borisova, Maxim Abakumov, Dmitry Gorin, Latchezar Avramov, Ivan Fedosov, Anton Namykin, Arkady Abdurashitov, Alexander Serov, Alexey Pavlov, Ekaterina Zinchenko, Vlad Lychagov, Nikita Navolokin, Alexander Shirokov, Galina Maslyakova, Dan Zhu, Qingming Luo, Vladimir Chekhonin, Valery Tuchin, Jürgen Kurths

**Affiliations:** ^1^Department of Physiology of Human and Animals, Saratov State UniversitySaratov, Russia; ^2^Huazhong University of Science and TechnologyWuhan, China; ^3^Laboratory of Biophotonics, Institute of Electronics, Bulgarian Academy of SciencesSofia, Bulgaria; ^4^Medico-Biological Department, Russian National Research Medical UniversityMoscow, Russia; ^5^Department of Nanotechnology, Saratov State UniversitySaratov, Russia; ^6^Department of Physics, Saratov State UniversitySaratov, Russia; ^7^Department of Electrical Engineering and Electronics, Saratov State Technical UniversitySaratov, Russia; ^8^Department of Pathological Anatomy, Saratov State Medical UniversitySaratov, Russia; ^9^Saratov Research Center, Institute of Biochemistry and Physiology of Plants and Microorganisms, Russian Academy of Sciences (IBPPM RAS)Saratov, Russia; ^10^Britton Chance Center for Biomedical Photonics, Wuhan National Laboratory for Optoelectronics, Huazhong University of Science and TechnologyWuhan, China; ^11^Laboratory of Biophotonics, Science Department, Tomsk State UniversityTomsk, Russia; ^12^Department of Physics, Humboldt UniversityBerlin, Germany; ^13^Research Domain Transdisciplinary Concepts and Methods, Potsdam Institute for Climate Impact ResearchPotsdam, Germany

**Keywords:** stress, newborn rats, cerebrovascular catastrophes, mechanisms

## Abstract

In this study, we analyzed the time-depended scenario of stress response cascade preceding and accompanying brain hemorrhages in newborn rats using an interdisciplinary approach based on: a morphological analysis of brain tissues, coherent-domain optical technologies for visualization of the cerebral blood flow, monitoring of the cerebral oxygenation and the deformability of red blood cells (RBCs). Using a model of stress-induced brain hemorrhages (sound stress, 120 dB, 370 Hz), we studied changes in neonatal brain 2, 4, 6, 8 h after stress (the pre-hemorrhage, latent period) and 24 h after stress (the post-hemorrhage period). We found that latent period of brain hemorrhages is accompanied by gradual pathological changes in systemic, metabolic, and cellular levels of stress. The incidence of brain hemorrhages is characterized by a progression of these changes and the irreversible cell death in the brain areas involved in higher mental functions. These processes are realized via a time-depended reduction of cerebral venous blood flow and oxygenation that was accompanied by an increase in RBCs deformability. The significant depletion of the molecular layer of the prefrontal cortex and the pyramidal neurons, which are crucial for associative learning and attention, is developed as a consequence of homeostasis imbalance. Thus, stress-induced processes preceding and accompanying brain hemorrhages in neonatal period contribute to serious injuries of the brain blood circulation, cerebral metabolic activity and structural elements of cognitive function. These results are an informative platform for further studies of mechanisms underlying stress-induced brain hemorrhages during the first days of life that will improve the future generation's health.

## Introduction

Cerebrovascular catastrophes, such as spontaneous brain hemorrhages in apparently normal full term newborns, are a real phenomenon. The problem is that in many cases different types and severity of brain hemorrhages in full term newborns are presented usually without any outward clinical symptoms or with any subtle non-specific neurological signs (Whitby et al., 2004; Looney et al., 2007; Rooks et al., 2008; Gupta et al., 2009). Therefore, the precise incidence and distribution in asymptomatic full term newborns is not known. However, during the last decade, improvement of neuroimaging technique made obvious that asymptomatic brain hemorrhages in full term newborns are frequent (from 26% till 46%) (Looney et al., 2007; Rooks et al., 2008). Notice, the majority of survived full term newborns after brain hemorrhages have a complete recovery (Jhawar et al., 2005; Siu et al., 2006; Rooks et al., 2008; Brouwer et al., 2010). But, neonatal death from brain hemorrhages can reach 25% due to asphyxia (Brouwer et al., 2010). The information about the neurological outcome after brain hemorrhages in full term newborns is extremely limited and typically is focused on the study of relatively short term follow-up with most babies not reaching school-age when higher order deficits are manifest (Jhawar et al., 2005; Siu et al., 2006; Rooks et al., 2008; Brouwer et al., 2010; Takenouchi et al., 2012; Kirton and deVeber, 2013). The results of few studies in this area showed that in future life such babies in 32% of cases develop cognitive deficit (Jhawar et al., 2005) and encephalopathy (Takenouchi et al., 2012), 21%—epilepsy (Siu et al., 2006), 14%—speech delay (Rooks et al., 2008), 8%—cerebral palsy (Brouwer et al., 2010). It is important to note that a child's brain develops over many years. As a result, difficulties can only be detected at an age when it is expected the development of relevant skills. For example, associative learning and thinking might not be recognized for many years. Therefore, the true incidence of cognitive impairment in asymptomatic neonates with brain hemorrhages may be slightly higher than they reported (Semyachkina-Glushkovskaya et al., 2016).

Thus, spontaneous brain bleeding in neonates is a major problem of future generation's health due to the high rate of death and cognitive disability of such newborns. Therefore, the study of mechanisms underlying silent pathological processes preceding and accompanying brain hemorrhages in neonatal period is absolutely essential.

In the majority, the reasons for brain hemorrhages in newborns cannot be found (Gupta et al., 2009). One possible factor is assisted delivery (Benedetti, 1999; Towner et al., 1999) (forceps or vacuum extraction), but those suggestions are not consistent (Whitby et al., 2004; Looney et al., 2007; Rooks et al., 2008). Blood clotting disorders may play an important role in brain bleeding causes in neonatal period (Gupta et al., 2009). However, there are no strong evidences in this detection (Gover et al., 2011). The major risk factor for newborns is stress, which babies have during embryonic development due to different stresses of mother, critical physiological changes during delivery and intensive adaptation to the new conditions of life. The first 3 days of life are the most critical. Mortality is highest in the initial 24 h after birth, up to 50% die within the first 3 days of life, and about 75% of all neonatal deaths occur in the first week of life (early neonatal death; Paul, 2006; Lawn et al., 2010). It is well-known that the neonatal environment is an important determinant of stress-related diseases (Maccari et al., 2003; Mirescu et al., 2004). However, stress itself and mechanisms underlying stress-related brain injury in neonates are not well-studied due to methodological difficulties.

Aiming to achieve a better understanding of mechanisms underlying stress-induced brain hemorrhages in neonatal period, in this experimental study on newborn rats, we intend to uncover a time-depended scenario of stress response cascade preceding and accompanying brain hemorrhages using interdisciplinary approach based on a morphological analysis of brain tissues, coherent-domain optical technologies for visualization of the cerebral blood flow (CBF), monitoring of the cerebral oxygenation and deformability of red blood cells (RBCs).

## Methods

### Subjects

Experiments were carried out in newborn mongrel rats, 12 days old using three groups: (1) intact, unstressed newborn rats in the control group (*n* = 25); (2) stressed rats in the pre-hemorrhage groups (2, 4, 6, 8 h after stress, *n* = 25 in each group); (3) stressed rats in the post-hemorrhage group (24 h after stress, *n* = 27). All procedures were performed in accordance with the “Guide for the Care and Use of Laboratory Animals.” The experimental protocol was approved by the Committee for the Care and Use of Laboratory Animals at Saratov State University (Protocol H-147, 17.04.2001).

Note, that the rat is a good animal subject for the study of the development of cerebrovascular catastrophes during the first days of life due to similar dynamics of the brain maturation in humans (Coyle, 1977). Our choice of the age of newborn rats is caused by the fact that at postnatal 12 days the brain of rats is close to the development of the brain in full-term human neonates (Rice et al., 1981).

### Modeling of brain hemorrhages

To induce brain hemorrhages, the following protocol of sound stress's impact was used (120 dB, 370 Hz): 10 s of sound followed by a 60-s pause; this cycle was repeated throughout a 2 h period (Semyachkina-Glushkovskaya et al., 2013, 2015a,b).

To analyze the brain-injures induced by stress and to confirm the development of brain hemorrhages, all newborn rats were decapitated for a histological study of brain tissue. The samples were fixed in 10% buffered neutral formalin. The formalin fixed specimens were embedded in paraffin, sectioned (4 μm), and stained with haematoxylin and eosin.

### State-of-the-art interdisciplinary approaches

**Figure 1:**
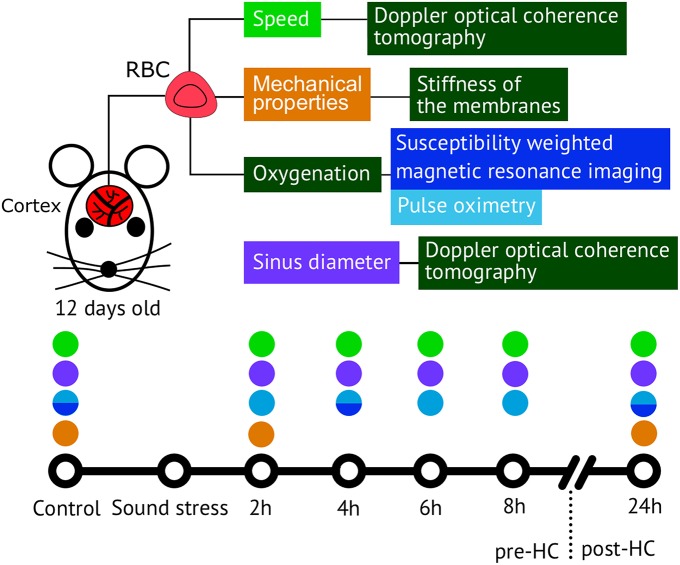
**The methods used for analysis of the time-depended scenario of stress response cascade preceding and accompanying brain hemorrhages in newborn rats 12 days old**.

To study the time-depended scenario of stress response cascade preceding and accompanying brain hemorrhages in newborn rats we used an interdisciplinary approach based on a morphological analysis of brain tissues, coherent-domain optical technologies for visualization of the cerebral blood flow, monitoring of the cerebral oxygenation, and the deformability of red blood cells (RBCs; Figure 1).

### Measurement of cerebral blood flow

To assess stress-related changes in cerebral circulation, we used a commercial swept source Doppler optical coherence tomography (DOCT) system OCS1300SS (Thorlabs Inc. USA) operating at 1325 nm central wavelength and 100 nm bandwidth. Transverse and axial resolutions of the DOCT system are 25 and 12 μm (on the air) respectively. A-scan rate is equal to 16 kHz, which allows us to measure absolute velocities up to ~5.5 mm/s (You et al., 2014).

### Analysis of cerebral oxygen saturation

The level of blood oxygen saturation (SpO_2_) in the brain as an important criterion of cerebral metabolic activity of the different functional states of an organism (Liu et al., 2015) was monitored by using the pulse oximeter model CMS60D (Contec Medical Systems Co., Ltd., Qinhuangdao, China). The optical sensor was based on dual wavelengths pulse oximetry approach, using 660 and 880 nm for the SpO_2_ detection. The oxy-hemoglobin saturation (SpO_2_) is given as a percentage of HbO_2_ vs. the total Hb in the blood. For confirmation of oximetry results, we used magnetic resonance imaging (MRI, Clin Scan 7T, Bruker Biospin) in susceptibility weighted regime (SWI) for determination of oxygenation of the brain tissues in the pre-hemorrhage (4 h after stress, *n* = 10) and the post-hemorrhage (24 h after stress, *n* = 10) groups in comparison with the control group (*n* = 10). SWI is a novel technique that is highly sensitive to local field in homogeneities and venous deoxygenated blood (Tsui et al., 2009).

### Evaluation of red blood cell deformability

Micropipette aspiration method was used to measure the resistance to deformation or stiffness of the membranes of red blood cells. It is based on measurement of RBC aspiration depth at given negative pressure applied to a micropipette with internal diameter of 2–2.5 μm (Mitchison and Swann, 1954; Rand and Burton, 1964; Artmann et al., 1997; Sinha et al., 2015; Zheng et al, 2015). Homemade glass micropipettes with 2 μm internal diameter were used for measurements. Pipettes were pulled using 0.93 mm borosilicate glass capillaries washed with isopropanol and distilled water. Aspiration procedure was imaged with inverted microscope using oil immersion lens (100 × NA = 1.25) and color CMOS camera (DCC1615C, Throlabs, Germany) to acquire RBC image. Pressure was applied using U manometer filled buffer solution and connected to the pipette. Blood samples for micropipette aspiration were diluted in 400 times with buffer solution. Aspiration depth ΔX was measured in equilibrium conditions. The goal of our study was to assess resistance to deformation for maximal possible number of cells in each sample. To reduce time required for individual measurement we determined only the deformation (delta x) of cell when constant low pressure is applied. After the negative pressure was applied to cell the aspirated part of membrane quickly reaches the equilibrium state when it no longer shifts along the pipette. Delta X was measured after membrane displacement rate decreases below one pixel (50 nm) for 30 s. It enables fast (2–3 min per cell) measurements of 30 cell within 1.5 h after blood sample preparation. That is sufficiently faster than measurements of critical pressure needed for estimation of membrane tension (Sinha et al., 2015).

### Statistical analysis

The results were presented as mean ± standard error of the mean (SEM). Differences from the initial level in the same group were evaluated by the Wilcoxon test. Intergroup differences were evaluated using the Mann-Whitney test and ANOVA-2 (*post-hoc* analysis with the Duncan's rank test). The significance levels were set at *p* < 0.05 for all analyses.

## Results

### Changes in the cerebral venous hemodynamics preceding and accompanying stress-induced brain hemorrhages in newborn rats

The vascular system is one of the first, which responds to stress to provide an adequate metabolism during the mobilization of the organism. However, in the neonatal period the vascular sensitivity to stress is different between perfusion (microcirculatory) and capacitive (venous) sectors of the cerebral circulation. In our previous study on newborn rats, we showed that the stress-reactivity of cerebral veins is higher than that of microvessels due to the specific function of veins to immediately change blood volume during stress and to maintain filling pressure to the heart (Semyachkina-Glushkovskaya et al., 2015a). Here we focused on the study of dynamic changes in the venous component of the cerebral circulation preceding and accompanying stress-induced hemorrhages in newborn rats. For this purpose, we selected the sagittal sinus that is one of major sinuses collecting blood from the small veins of the brain and directs it into the peripheral circulation. We choose this vessel to perform measurements of hemodynamic parameters through the anterior fontanel as a window to the brain in newborn animals.

**Figure 2:**
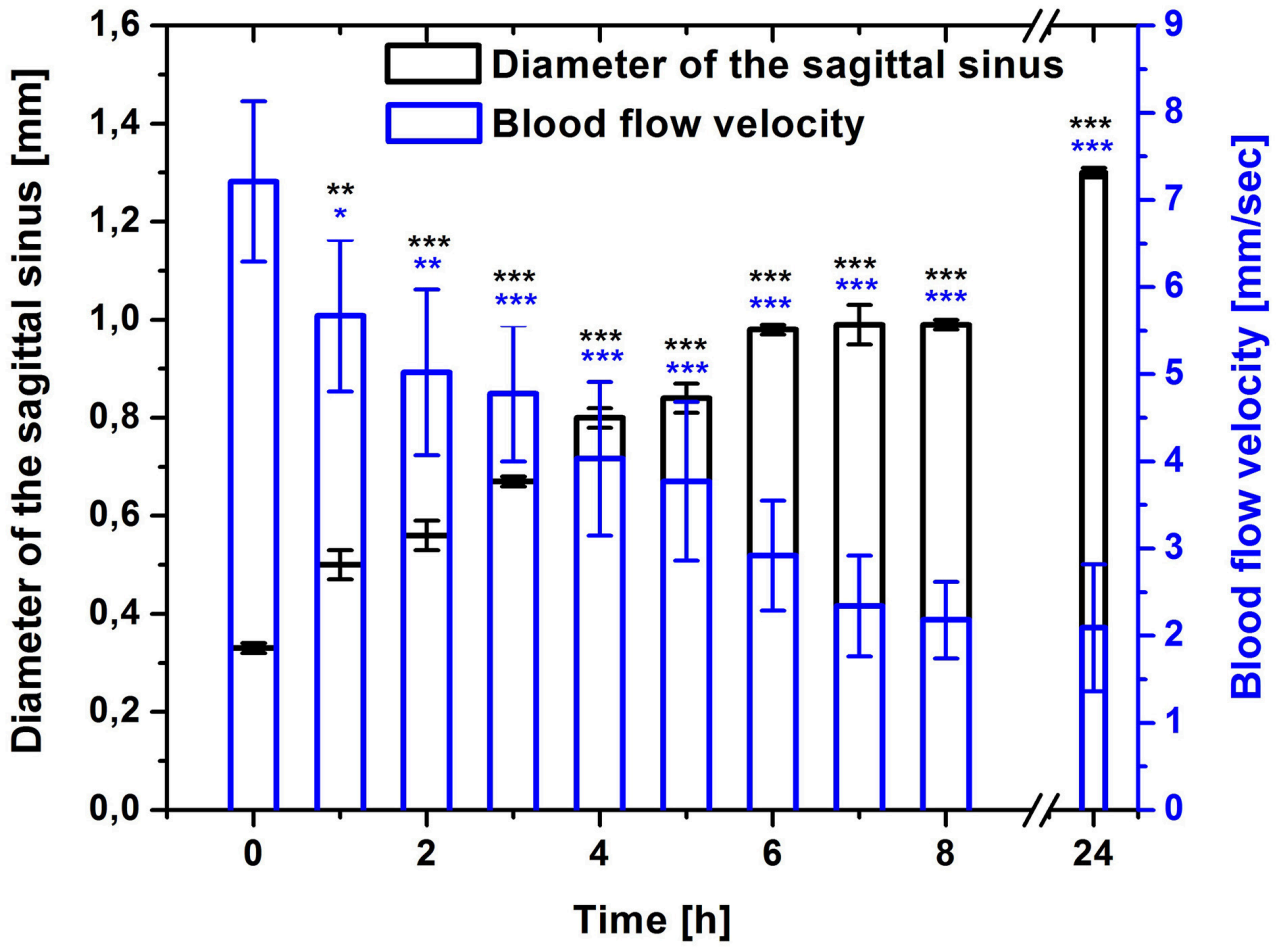
**Time-dependent changes in the sagittal sinus of newborn rats (diameter, mm and blood flow velocity, mm/s) in the pre-hemorrhage (2, 4, 6, 8 h after stress, ***n*** = 25 in each group) and post- hemorrhage (24 h after stress, ***n*** = 27) groups in comparison with control group (***n*** = 25)**. ^*^P < 0.05; ^**^P < 0.01; ^***^P < 0.001 compared with the control values (0 h).

Figure 2 shows the DOCT results, which demonstrate time-depended changes in diameter of the sagittal sinus and blood flow velocity before and after stress-induced brain hemorrhages in newborn rats. During the pre-hemorrhage period (2, 4, 6, and 8 h after stress), we observed a increase in the size of the sagittal sinus and a gradual decrease in the blood flow velocity. The maximal changes of these hemodynamic parameters were observed during the incidence of brain hemorrhages (24 h after stress).

### Changes in the blood oxygen saturation of the brain tissues and erythrocyte deformability preceding and accompanying stress-induced brain hemorrhages in newborn rats

Although investigators have implicated hypoxia as a potential risk factor for the brain hemorrhages in neonates (Michoulas et al., 2011; Luo et al., 2014; van der Aa et al., 2014), the role of hypoxia in the intracranial hemorrhages remains controversial because brain bleeding itself may cause respiratory distress. Therefore, it is difficult to ascertain, if cerebral hypoxia is a key factor in brain hemorrhages, or if it is a consequence of this condition (Jhawar et al., 2003). For a better understanding of the role of hypoxia in the development of brain hemorrhages in the neonatal period, at the second step of our work, we studied the level of SpO_2_ in the brain of newborn rats. With this aim, we used the pulse oximetry that confirmed by MRI-SWI imaging.

**Figure 3:**
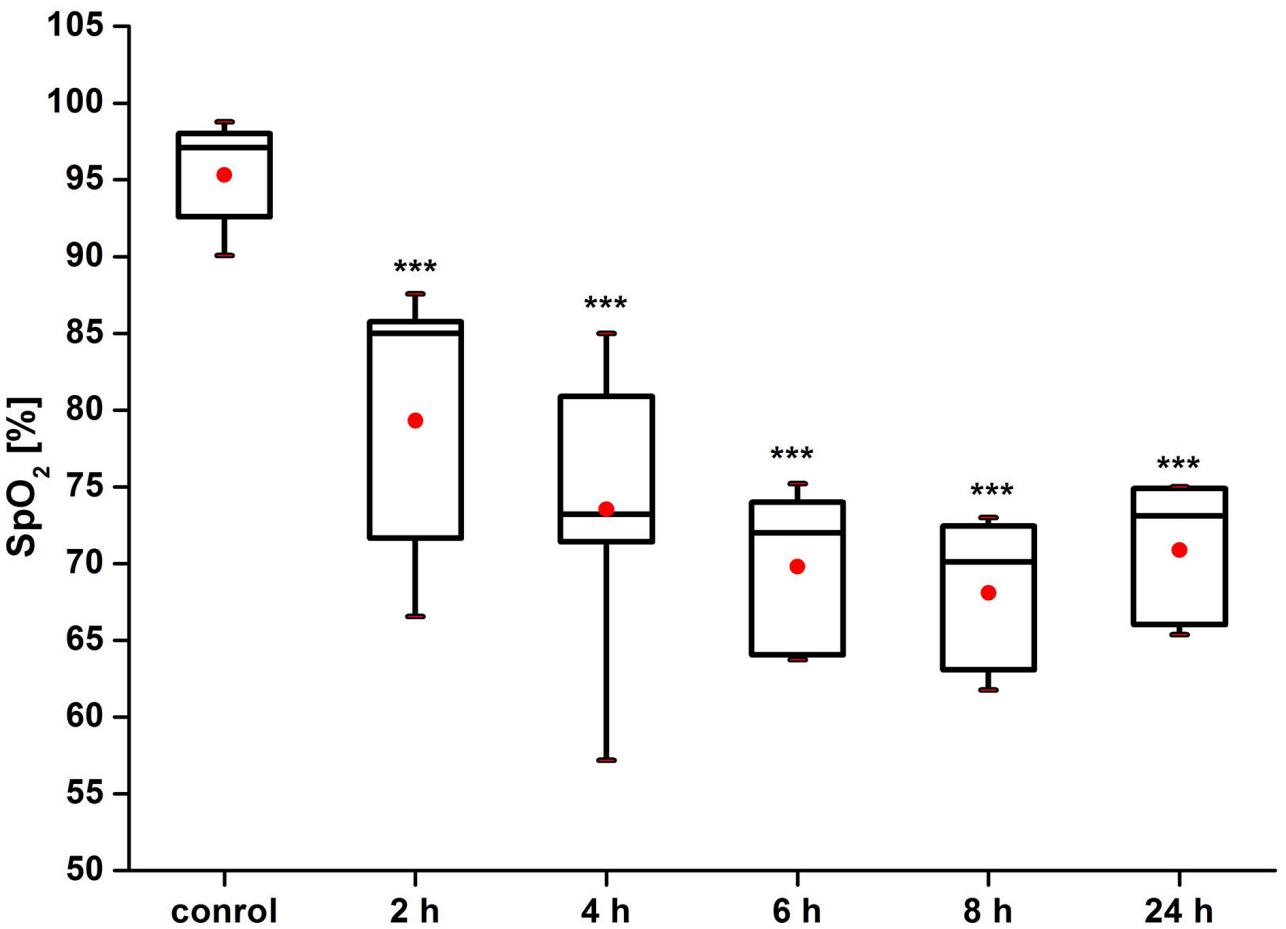
**Time-dependent changes in blood oxygen saturation of the brain of newborn rats in the pre-hemorrhage (2, 4, 6, 8 h after stress, ***n*** = 25 in each group) and post- hemorrhage (24 h after stress, ***n*** = 27) groups in comparison with control group (***n*** = 25)**. ^***^P < 0.001 compared with the control values.

Figure 3 shows that the pre-hemorrhage group (*n* = 15) demonstrated a gradual reduction of the SpO_2_ level. Indeed, the decrease in SpO_2_ was observed during the whole time before the incidence of brain hemorrhages with maximal reduction of oxygen delivery 4–8 h after stress (by 27, 28, 30, respectively; *p* < 0.05). The post-hemorrhage group showed the decrease in SpO_2_ level by 27% (*p* < 0.05), i.e., it remained low.

**Figure 4:**
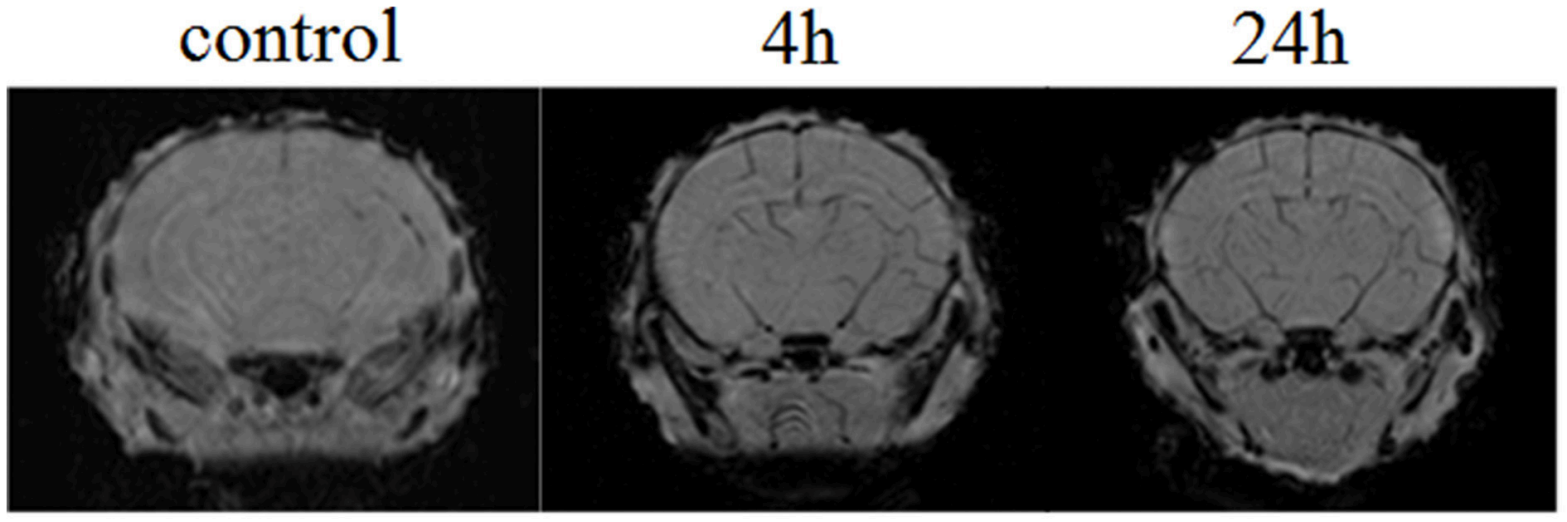
**The example of MRI-SWI image of neonatal rat brain in normal state (the control group, ***n*** = 10), in the pre-hemorrhage period (4 h after stress, ***n*** = 10) and post-hemorrhage period (24 h after stress, ***n*** = 10)**.

Figure 4 demonstrates the example of MRI-results in SWI regime obtained from 10 newborn rats in each group (the control, pre- and post-hemorrhage). The pre- and post-hemorrhage periods were characterized by hypointense contrasting of cerebral vessels. These changes can be caused by the accumulation of deoxyhemoglobin due to prolonged hypoxia leading to reduced oxygen levels.

The diminished oxygen delivery to the brain tissues in response to a hypoxia may be related to changes of RBC mechanical properties (Ellsworth et al., 1995; Sprague et al., 1996, 2001, 2003; Parthasarathi and Lipowsky, 1999; Kirby et al., 2012). However, the information about the role of erythrocyte deformability in hypoxia after brain hemorrhages is significantly limited (Pollock and Harrison, 1982; Grotta et al., 1986). The question about the pre- and post-hemorrhage changes in the RBCs elasticity during hypoxia remains open. We assumed that the changes of RBC deformability might be one of mechanisms responsible for hypoxia in stressed newborn rats. To check our hypothesis we analyzed RBC deformability in newborn rats before and after stress-induced hemorrhages using the method of micropipette aspiration. We chose two important points for experiment: 2 h after stress when the first decrease in SpO2 occurred in the pre-hemorrhage group (*n* = 15) and 24 h after stress in the post-hemorrhage group (*n* = 15).

**Figure 5:**
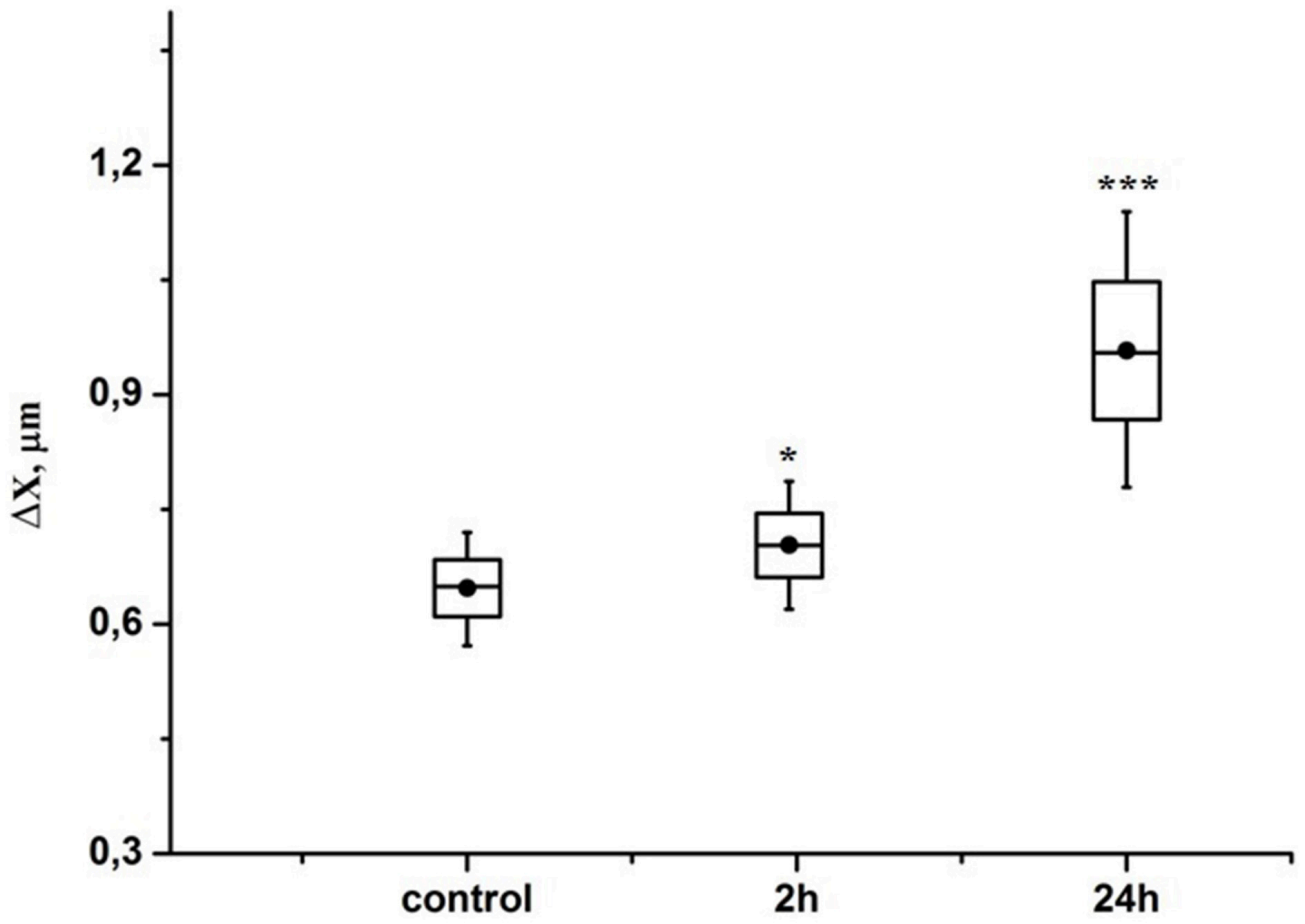
**Time-dependent changes in the aspiration depth of red blood cells in the pre-hemorrhage (2 h after stress, ***n*** = 25) and post- hemorrhage (24 h after stress, ***n*** = 27) groups in comparison with control group (***n*** = 25)**. Figure shows aspiration depth at 30 N/m^2^ pressure measured in blood samples taken in different time interval after stress. Each point corresponds to a value averaged over measurements performed with 30 different RBCs. ^*^P < 0.05; ^***^P < 0.001 compared with the control values.

Figure 5 demonstrates that the RBCs deformability increased by 10% (*p* < 0.05) in the pre-hemorrhage group and by 40% (*p* < 0.001) in the post-hemorrhage group.

### Changes in the brain areas involved in higher cognitive functions before and after the stress-induced brain hemorrhages in newborn rats

The prefrontal cerebral cortex is a special zone of the brain, which includes the higher-order association areas and plays a key role in memory, attention, perception, awareness, thought, language, and consciousness. The cognitive deficit in babies is highly associated with brain hemorrhages, which they had in neonatal period in the frontal lobe (Jhawar et al., 2005; Rooks et al., 2008; Semyachkina-Glushkovskaya et al., 2016). However, the mechanisms underlying these pathological processes remain poorly understood and request detailed studies in this field. With this aim, at the final step of our work, we studied the morphological changes in the molecular layer of the prefrontal cortex and in the pyramidal neurons, which are crucial for the “feedback” interactions in the cerebral cortex involved in associative learning and attention (Gilbert and Sigman, 2007).

**Figure 6:**
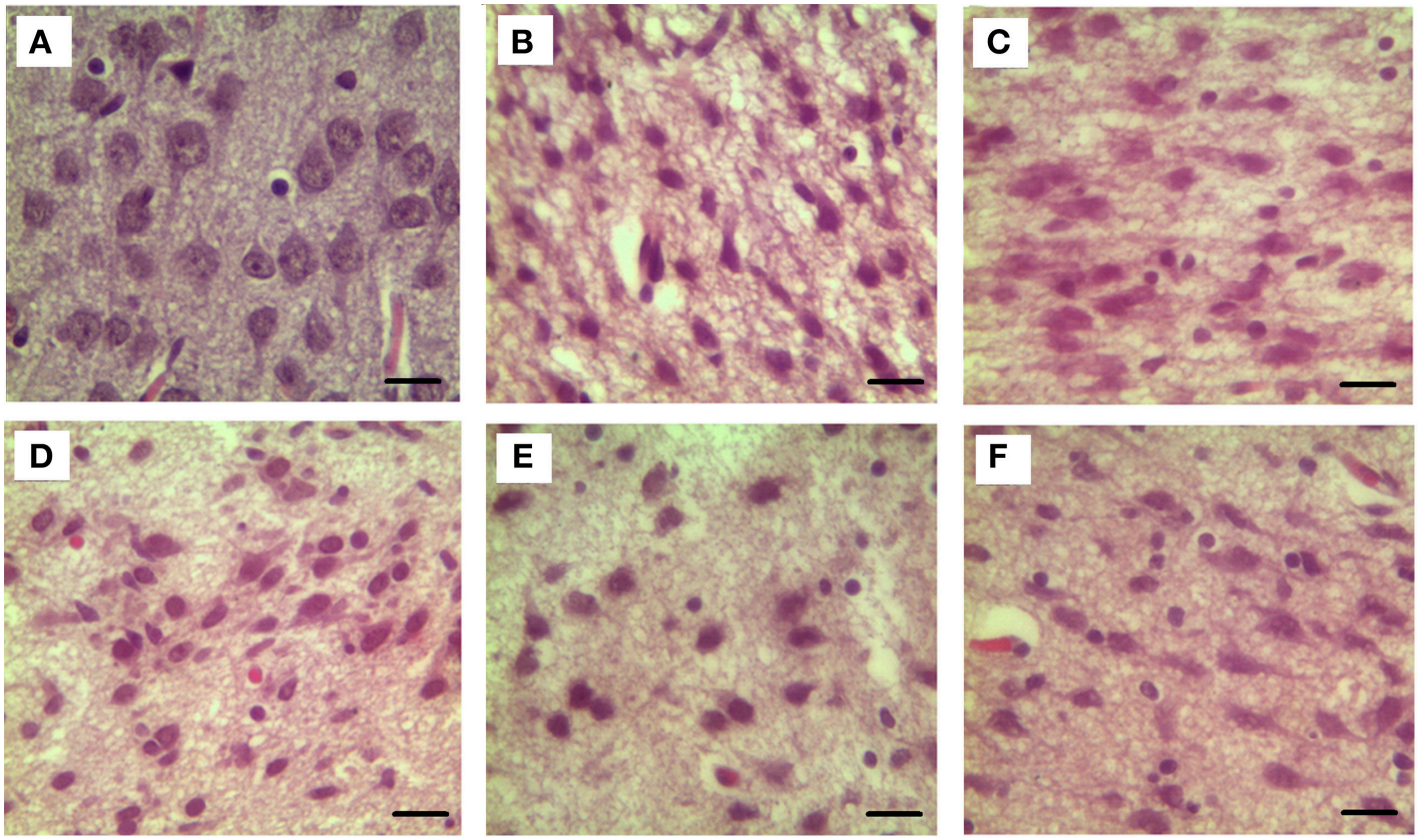
**The time-depended changes in the diameter of pyramidal neurons of newborn rats in the pre-hemorrhage (2, 4, 6, 8 h after stress, ***n*** = 25 in each group) and post- hemorrhage (24 h after stress, ***n*** = 27) groups in comparison with control group (***n*** = 25)**. Hematoxylin and Eosin staining. Bars represent 10 μm (774X). **(A)**—the normal state (the control); the pre-hemorrhage period (**B–E**—2, 4, 6, 8 h after stress); the post-hemorrhage period (**F**—24 h after stress).

**Figure 7:**
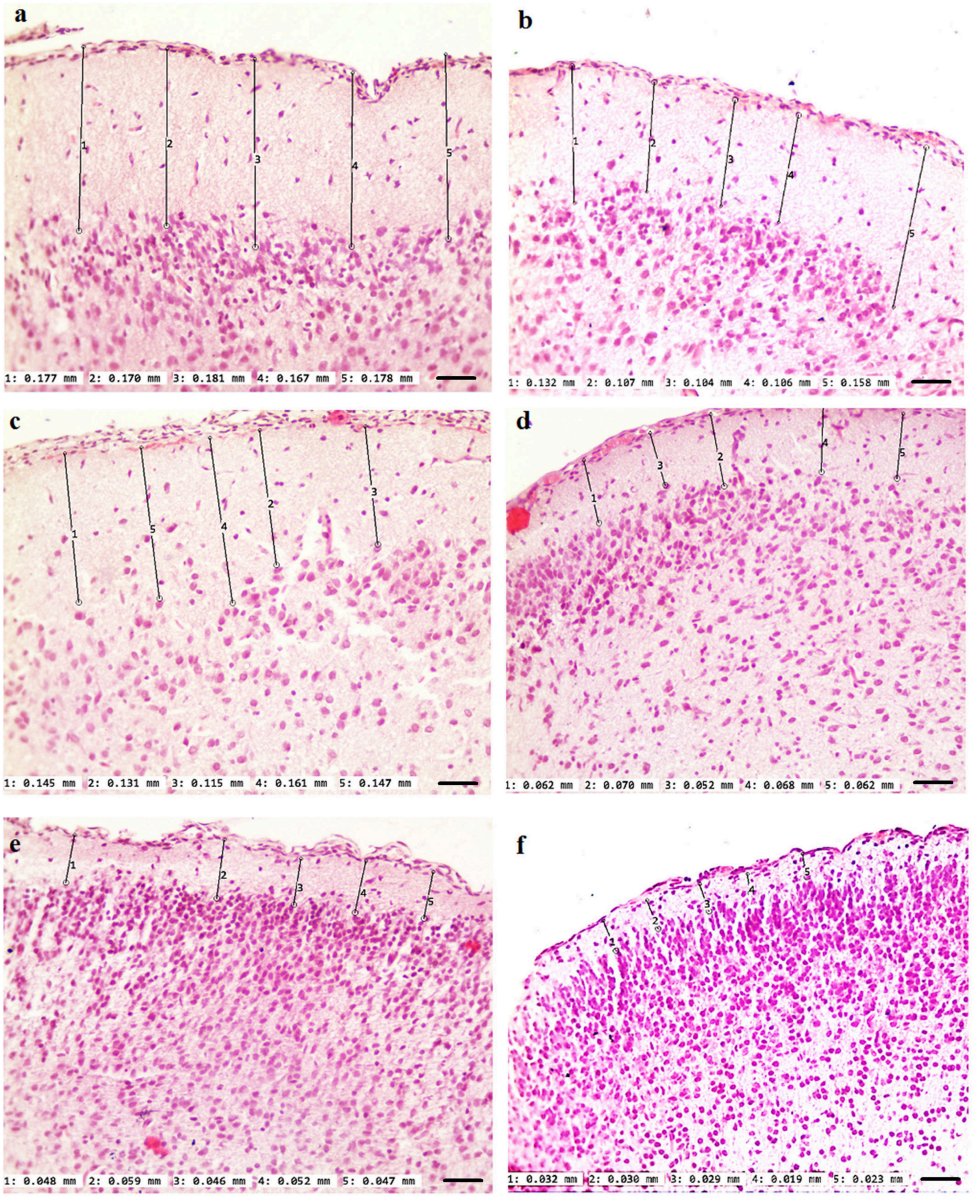
**The time-depended changes in the thickness of the molecular layer of the prefrontal cortex of newborn rats in the pre-hemorrhage (2, 4, 6, 8 h after stress, ***n*** = 25 in each group) and post-hemorrhage (24 h after stress, ***n*** = 27) groups in comparison with control group (***n*** = 25)**. Hematoxylin and Eosin staining. Bars represent 10 μm (246X). **(A)**—the normal state (the control); the pre-hemorrhage period (**B–E**—2, 4, 6, 8 h after stress); the post-hemorrhage period (**F**—24 h after stress).

**Figure 8:**
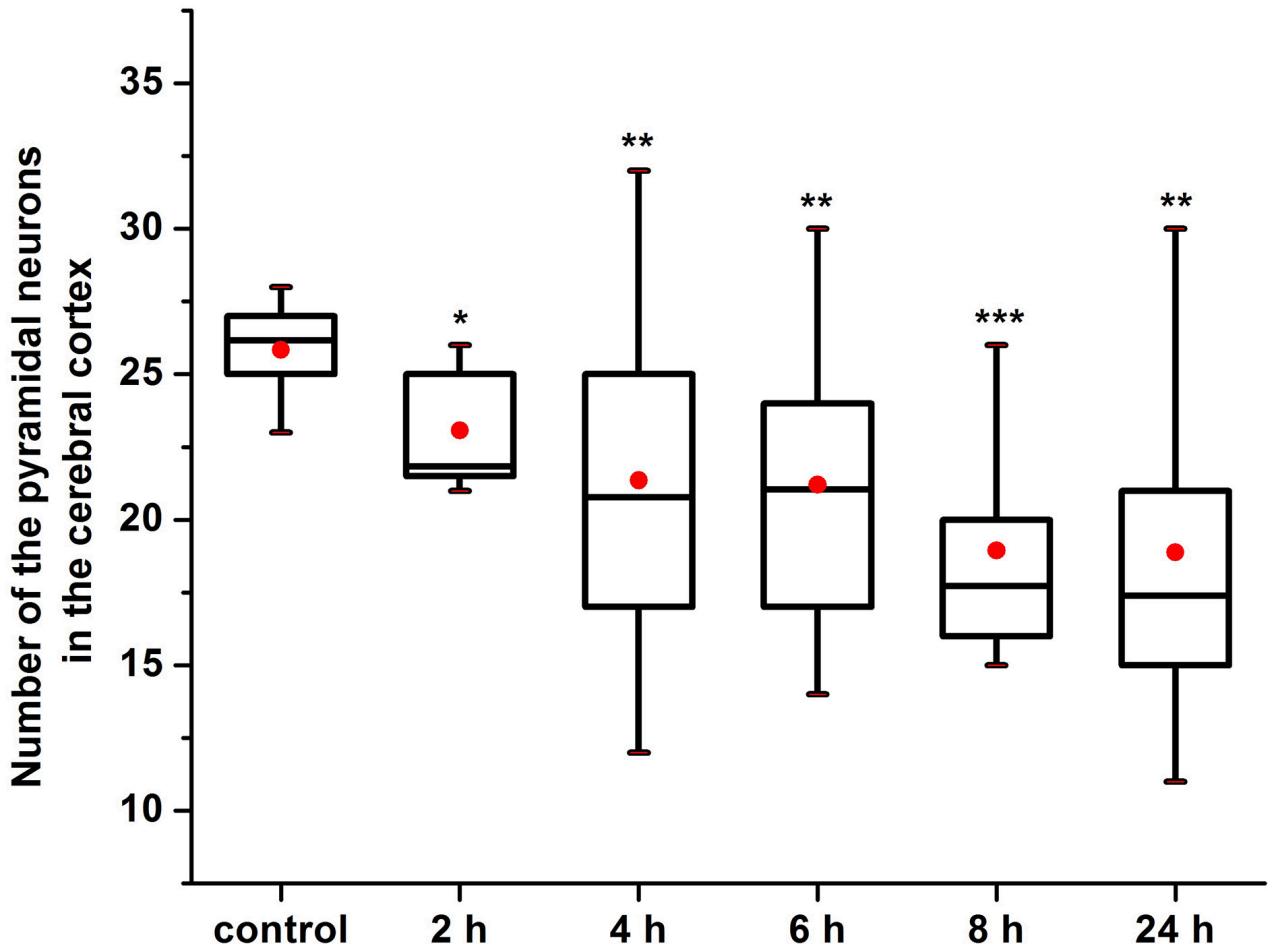
**The time-depended changes in the number of pyramidal neurons in newborn rats in the pre-hemorrhage (2, 4, 6, 8 h after stress, ***n*** = 25 in each group) and post- hemorrhage (24 h after stress, ***n*** = 27) groups in comparison with control group (***n*** = 25)**. ^*^P < 0.05; ^**^P < 0.01; ^***^P < 0.001 compared with the control values.

**Figure 9:**
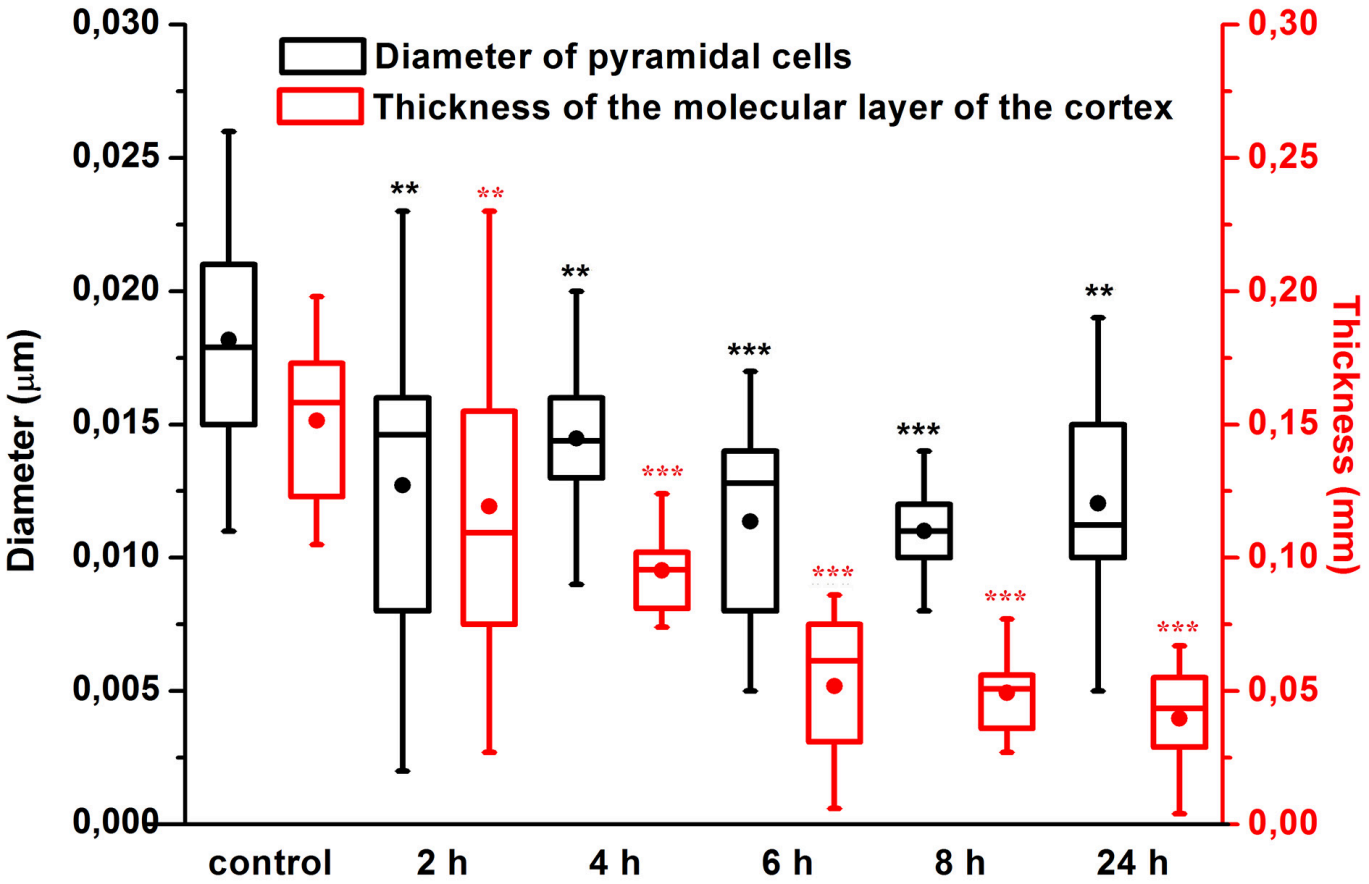
**The time-depended changes in the thickness of the molecular layer of the prefrontal cortex and in the diameter of pyramidal neurons in newborn rats in the pre-hemorrhage (2, 4, 6, 8 h after stress, ***n*** = 25 in each group) and post-hemorrhage (24 h after stress, ***n*** = 27) groups in comparison with control group (***n*** = 25)**. ^*^P < 0.05; ^**^P < 0.01; ^***^P < 0.001 compared with the control values.

**Figure 10:**
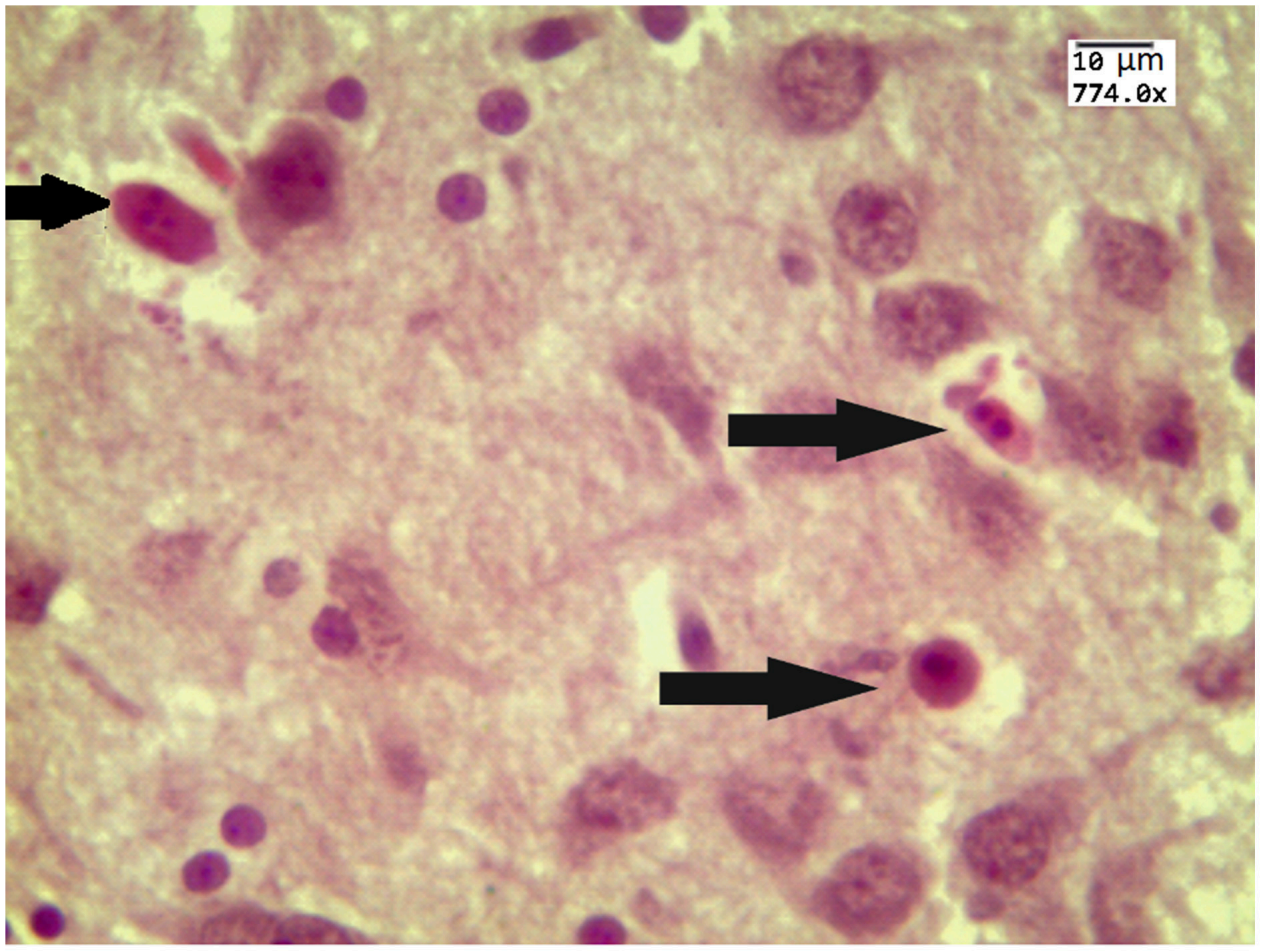
**The example of apoptotic bodies (arrowed) in the prefrontal cortex obtained from 27 newborn rat 24 h after stress (the signs of apoptosis are condensation and fragmentation of nuclei in an intensely eosinophilic cytoplasm)**. Hematoxylin and Eosin staining. Bars represent 10 μm (774X).

**Figure 11:**
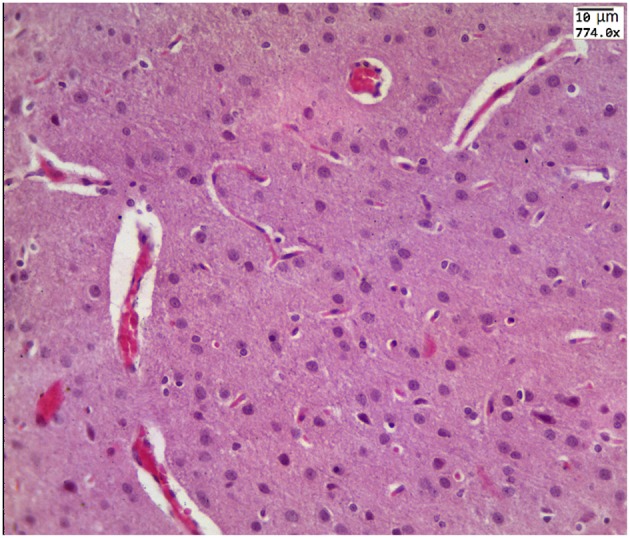
**The example of perivascular edema i.e., fluid pathway from the cerebral vessels (arrowed) in the brain obtained from the pre-hemorrhage group (2, 4, 6, 8 h after stress, ***n*** = 25 in each group) and in the post-hemorrhage group (24 h after stress, ***n*** = 27)**.

Our results clearly show that the pre-hemorrhage group (*n* = 15) demonstrated a gradual decrease in the thickness of the molecular layer of the cortex and in the number of pyramidal neurons with reducing of their diameter (Figures 6–9). So, during the pre-hemorrhage period (2, 4, 6, and 8 h after stress) the thickness of the molecular layer of the cortex was decreased by 31, 40, 62, and 68%, respectively, *p* < 0.05; the number of pyramidal cells—by 17, 21, 21, and 33%, respectively, *p* < 0.05; the diameter of pyramidal neurons—by 19, 20, 29, and 39%, *p* < 0.05, respectively (Figures 8, 9). The incidence of the brain bleeding was accompanied by a more pronounced decrease in the thickness of the molecular layer of the cortex, which was reduced by 73% (*p* < 0.05) compared with the normal state. The number of pyramidal cells and their diameter were decreased by 34% (*p* < 0.05) and 38% (*p* < 0.05), respectively, i.e., it remained reduced. These pathological changes in the cortex were accompanied by the apoptosis of pyramidal cells (Figure 10). Furthermore, we observed the development of the perivascular edema during all pre- and post-hemorrhages time in all newborn rats (Figure 11). But, there were no any stressful changes in severity of the fluid pathway from the cerebral vessels in the pre- and post-hemorrhage groups.

The results of three series of experiments suggest that the reduction of blood circulation in the cerebral venous system and hypoxia are the important factors for disorders of the functional platform for the integration of brain centers, such as the molecular layer of the cortex and the pyramidal neurons.

## Discussion

In this interdisciplinary study we analyzed the time-dependent scenario of stress-induced brain hemorrhages in newborn rats on the different levels of cascade of stress reaction on morphological (histological analysis of cerebral cortex), systemic (monitoring of cerebral venous blood flow), metabolic (assessment of oxygen saturation of the brain tissues), and cellular (changes in RBCs deformability and pyramidal neuron number and diameter) levels.

In our previous studies, we clearly showed the location, types, depth, and morphological changes related to stress-induced brain hemorrhages in newborn rats (Semyachkina-Glushkovskaya et al., 2015a,b). Based on our earlier studies, we suggest that the venous component of the cerebral circulation is highly sensitive to the stress in the neonatal period (Semyachkina-Glushkovskaya et al., 2013, 2015a; Pavlov et al., 2014). Here we focused on the study of time dynamics of changes in the cerebral venous system preceding and accompanying stress-induced brain hemorrhages in newborn rats. The object of our study was the sagittal sinus, which collects blood from all veins of the brain and directs it into the peripheral circulation. DOCT imaging uncovers time-dependent progressive changes in this vessel during the pre-hemorrhage time (2, 4, 6, and 8 h after stress) and after incidence of brain bleeding (24 h after stress). The latent period of brain hemorrhages is characterized by a relaxation of the sagittal sinus with a fall of blood flow velocity in it that was more pronounced after incidence of brain bleedings in newborn rats. Notice, the changes in these two hemodynamic parameters are closely not correlated. We observed the gradual reduction of venous blood flow during all pre- and post-hemorrhage time, while the increase in the diameter of sagittal sinus was from 6 her till 8 her after stress stable. But, 24 h after stress the sagittal sinus dilated significantly compared with other periods of observations. These changes can be explained by mechanisms underlying distributions of cerebral blood flow under stress. In our previous work we showed that the progressive relaxation of cerebral veins in newborn rats with the stroke causes accumulation of blood not only in venous network but also in microvessels due to the redistribution of blood flow in the cerebral vessels to decrease the pressure of accumulated blood on the thick walls of cerebral veins (Semyachkina-Glushkovskaya et al., 2015a).

We also observed that the pathological relaxation of the main cerebral vein was accompanied by the formation of perivascular edema i.e., fluid pathway from the vessels. In our previous work, the histological data show that the increase in size of the sagittal sinus is associated with the dilation of all cerebral veins, especially in the pail matter of the cerebral cortex (Semyachkina-Glushkovskaya et al., 2015a). The relaxation of cerebral veins with perivascular edema is a marker of accumulation of an extensive blood in the venous system and suppression of blood outflow from the brain leading to venous insufficiency (Valdueza et al., 2013).

Thus, the sagittal sinus shows sensitive changes to the deleterious effect of stress from the pre-hemorrhage time until the incidence of brain bleeding. Clinical studies also have shown that neonatal intracranial hemorrhages are primary venous infarction due to a weakness of the wall of cerebral veins in neonates (Hambleton and Wigglesworth, 1976; Ghazi-Birry et al., 1997; Bruno et al., 2014).

A gradual reducing of the blood flow velocity in the dilated sagittal sinus in stressed newborn rats is associated with a time-dependent reduction of oxygen supply to the brain. The results of our experiments clearly show that cerebral hypoxia precedes stress-induced brain hemorrhages. This fact is consistent with other experimental and clinical data. Thoresen et al. in experiments on newborn pigs demonstrated that severe hypoxia itself can induce spontaneous brain hemorrhage in newborn pigs (Thoresen et al., 2001). Aderliesten et al. in clinical observations showed a lower cerebral fraction tissue oxygen extraction in newborns before severe stroke (Alderliesten et al., 2013). Taking into account these facts we suppose that hypoxia in the pre-hemorrhage period might be a causative factor provoking critical changes in the brain, associated with intracerebral hemorrhage in newborn rats.

Our results clearly exhibit that the cerebral hypoxia and disturbances in the cerebral venous system in newborn rats are accompanied by an increase in deformability of RBCs. The deformation of RBCc is a key factor for a release of powerful vasorelaxant such as adenosine 5′ triphosphate (ATP) (Sprague et al., 1996). This mechanism is related to the activation by β-adrenergic receptors presented at the RBCs' surface (Olearczyk et al., 2001). In our previous study, we showed the high sensitivity of cerebral vessels of newborn rats to a pharmacological modulation of vascular β-adrenergic receptors (Pavlov et al., 2014). These facts allow us to believe that the increased deformability of RBCs in stressed newborn rats might be one of possible mechanisms contributing to the pathological relaxation of the cerebral veins due to ATP release.

A stress-induced gradual reduction of oxygen supply to the brain and blood flow velocity in the cerebral venous system were accompanied by gradual pathological changes in the brain areas involved in higher cognitive functions such as the molecular layer of the prefrontal cortex and the pyramidal neurons, which are crucial for associative learning and attention (Gilbert and Sigman, 2007). The severity of stress-related disorders on the level of cerebral venous circulation and oxygenation of the brain tissues was associated with the time-dependent decrease in the thickness of the molecular layer of the prefrontal cortex as well as with the reduction of the number of pyramidal neurons and their diameter. The formation of perivascular edema, which we observed in the pre- and post-hemorrhage periods, can be one of the reasons responsible for a decrease in the diameter of the pyramidal neurons due to the fluid pathway from the cerebral vessels and mechanical compression. Notice that the incidence of the brain hemorrhages was accompanied by the progression of pathomorphological changes in the cerebral cortex up to the apoptosis of pyramidal neurons, i.e., irreversible changes leading to the death of cerebral cells. In our previous morphological studies of the brain tissues and cerebral vessels in newborn rats we clearly show the progressive accumulation of blood in the superficial cerebral veins (the pre- hemorrhage time) and in deep cerebral veins and microvessels (the post-hemorrhage time) (Semyachkina-Glushkovskaya et al., 2015a). We assume that vascular component of stress-related brain injures play an important role in drastic shrinkage of the prefrontal cortex via mechanisms of compressive pressure on the molecular layer of the prefrontal cortex.

What does the brain hemorrhages mean for intellectual functions of the brain? The results of our study give clear evidence that even an early latent stage of brain bleeding is associated with significant morphological lesions of the “intellectual zone” of the prefrontal cortex that is accompanied by the irreversible apoptosis process after the incidence of brain hemorrhages. Thus, stress-induced processes preceding and accompanying brain hemorrhages in neonatal period contribute the serious injures of the brain association areas responsible for cognitive functions. Future studies need to focus on long-term outcomes after brain hemorrhages induced by stress in neonatal period to develop effective prognostic criteria and to optimize neuroprotective strategies.

## Conclusion

In general, our results suggest significant time-dependent stress-induced changes in the brain preceding and accompanying intracranial hemorrhages in newborn rats. The pre-hemorrhage processes are realized at different levels of stress response cascade such as systemic (the progressive reduction of blood flow circulation in the cerebral venous system), metabolic (the cerebral oxygenation declines), and cellular one's (the increase in RBCs deformability; the decrease in the thickness of the molecular layer in the prefrontal cortex and in the number of pyramidal neurons with reducing their diameter). The post-hemorrhage time is characterized by progression of stress-induced systemic, metabolic, and cellular changes in the brain, which are accompanied by irreversible cell death apoptosis process in the brain areas involved in higher cognitive functions. Thus, the stress-induced processes preceding and accompanying brain hemorrhages in the neonatal period contribute to serious injures of the brain blood circulation, cerebral metabolic activity and the structural elements of cognitive function. These results are an informative platform for further studies of mechanisms underlying stress-induced brain hemorrhages during the first days of life that will improve the health of the future generation.

## Author contributions

OS: wrote the text of article, analyzed all results. EB: performed a measurement of cerebral oxygenation. MA: performed monitoring of oxygen saturation using magnetic resonance imaging (MRI) of the brain of newborn rats. DG: prepared protocol of anesthesia for newborn rats during all experiments. LA: made analysis of the changes in the cerebral oxygenation. IF: developed the plan of optical experiments with the red blood cells deformability. AN: performed a measurement of red blood cells deformability. AA: performed a measurement of cerebral venous blood flow. AS: performed a model of stress-induced brain bleeding in newborn rats. AP: performed the statistical analysis of data. EZ: prepared animals for all experimental tasks. VL: analyzed the recording of DOCT data. NN: performed the histological analysis. GM: analyzed the histological data. DZ: gave consultation about a measurement of cerebral blood flow in newborn animals. QL: discussed DOCT results. VC analyzed MR data. VT: coordinated experiments with the coherent-domain optical technologies. JK: discussed results and application of interdisciplinary methods in the experiments. AS made algorithm of sound stress and selected suitable frequence of sound to induce brain hemorrhages in newborn rats. He also performed the final approval of article.

### Conflict of interest statement

The authors declare that the research was conducted in the absence of any commercial or financial relationships that could be construed as a potential conflict of interest.
